# A Higher Curriculum for Educated Probationers

**Published:** 1921-03-05

**Authors:** 


### A Higher Curriculum for Educated Probationers.

It is not only in England that the business of preparing nurses for everyday duties in private nursing and in institutions is found to conflict with the training of women prepared by a higher education to take organising and teaching posts. It is interesting to find that both in America and in Canada a movement is arising for differentiating two grades of nurses. Dr. Elisha Cahoon, in the Boston

*Medical and Surgical Journal* for January, argues against the proposals which are from time to time weakly urged in the United States for an inferior class of "licensed attendant" as a panacea for the shortage of nurses. But he is strongly in favour of a plan which admits of two grades, the first to consist of nurses who have taken at least a three or more years' course in a hospital of recognised standing and whose preliminary requirements should be a high school or college course. The second grade would have had at least a grammar-school education and at least eighteen months or more of good hospital training, the latter class to be known as registered junior nurses and be at liberty to advance to the higher grade. Such a proposal would find no supporters in this country, but it is at any rate an advance on the "licensed attendant" scheme.

In *The Canadian Nurse*, Miss Ethel Johns, director of nursing at the Vancouver General Hospital, tackles the same problem. She is being urged on all sides to lower standards, to shorten courses, to do, in short, something or anything to get nursing attendance for people who need it. "We are between the devil and the deep sea: we are asked to prepare in the same school and under the same conditions, and with the same methods, women for two distinct types of service—routine and highly skilled. The demand for highly trained women for public health, for training-school and hospital administration is overwhelming. There are on the book of Teachers' College three hundred requests for women possessing special training for which there is no supply, because women capable of assuming work of this kind are content to drift along in routine work which does not develop their real powers."

We are less scourged in this country with the demand for nurses to be made in a hurry. But we are definitely confronted with the challenge that our training-school methods often fail to bring out the real powers of the probationers, and that the more highly educated are sacrificed to the exigencies of preparing them side by side with women of less highly developed mental faculties for very easy examinations.

